# Knee Synovial Haemangioma: A Case Report and Imaging Perspective on Diagnosis

**DOI:** 10.5334/jbsr.3697

**Published:** 2024-11-25

**Authors:** Inês Da Mata, Ana Filipa Forjaco Jorge, Eduardo Bandeira, João Cabral Pimentel

**Affiliations:** 1 Department of Radiology, Unidade Local de Saúde São José (ULS São José), Lisbon, Portugal; 2 Department of Radiology, Unidade Local de Saúde São José, Lisbon, Portugal; 3 Service of Anatomic Pathology, Unidade Local de Saúde São José (ULS São José), Lisbon, Portugal

**Keywords:** Synovial haemangioma, synovial tumours, magnetic resonance imaging

## Abstract

Synovial haemangioma is a rare benign entity, most common in children and adolescents. These tumours can extensively infiltrate joint structures and periarticular soft tissues, making management challenging. Magnetic resonance imaging (MRI) has a key role in diagnosis and therapeutic planning. The authors report the case of a 17‑year‑old male with multiple synovial haemangiomas, highlighting the complexity of management and the essential role of MRI in assessment and treatment.

*Teaching point:* Synovial haemangiomas may lead to bone remodelling, periarticular soft‑tissue infiltration and, ultimately, limb deformities and early‑onset osteoarthritis. Magnetic resonance imaging (MRI) is crucial for diagnosis as well as for guiding therapeutic planning.

## Introduction

Synovial haemangioma (SH) is a rare benign tumour resulting from blood vessel proliferation in the synovial surface of joints, bursae and tendon sheaths [1, 2]. A recent review highlights the rarity of this condition, with most studies consisting of single clinical cases [3]. The knee is the most commonly affected joint [1–3]. SH is more frequent in children and young adults, typically presenting with recurrent joint pain and spontaneous swelling [1–3]. Histologically, SH is categorized into capillary, cavernous, arteriovenous and venous types on the basis of the size and nature of the vessels [1, 2]. Knee SH can present as diffuse, localized intra‑articular or juxta‑articular synovial masses, potentially leading to bone remodelling, cartilage damage and degenerative joint disease [1–3].

## Case Report

The authors present a case study of a 17‑year‑old male consulting regarding orthopaedic surgery due to progressive pain and swelling of the right knee resulting in functional impairment and ipsilateral muscular atrophy. The patient did not report any significant trauma or other relevant medical conditions. A total of 11 years earlier, the patient was diagnosed with synovial haemangioma of the right ankle, confirmed by excisional biopsy.

**Figure 1:**
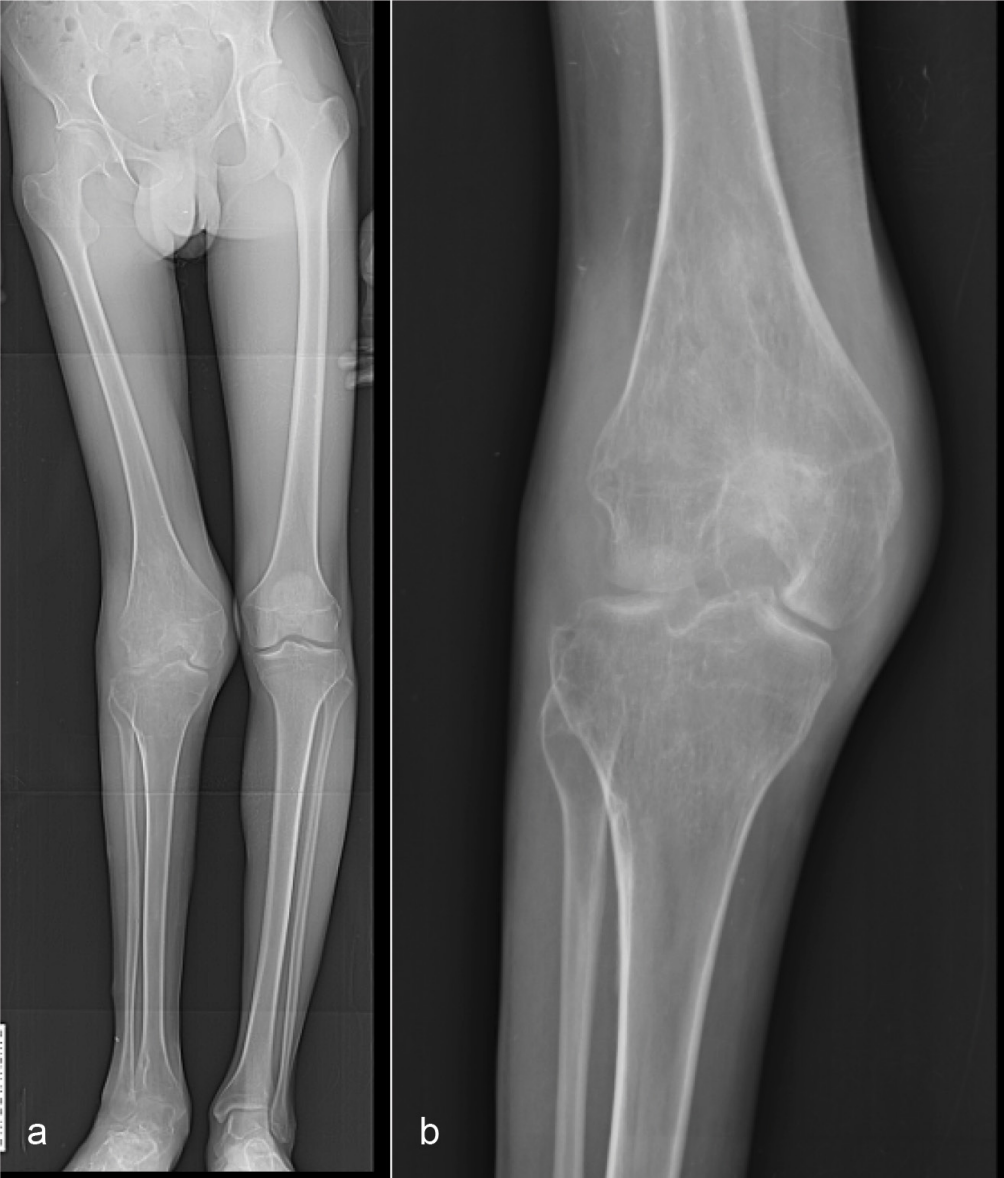
Full‑length radiograph **(A)** and right knee radiograph **(B)** show reduced bone mineralization, bone remodelling with intercondylar groove widening, epiphyseal overgrowth and valgus knee deformity. Secondary degenerative changes in all joint compartments are noted. No bone lesions were detected.

**Figure 2:**
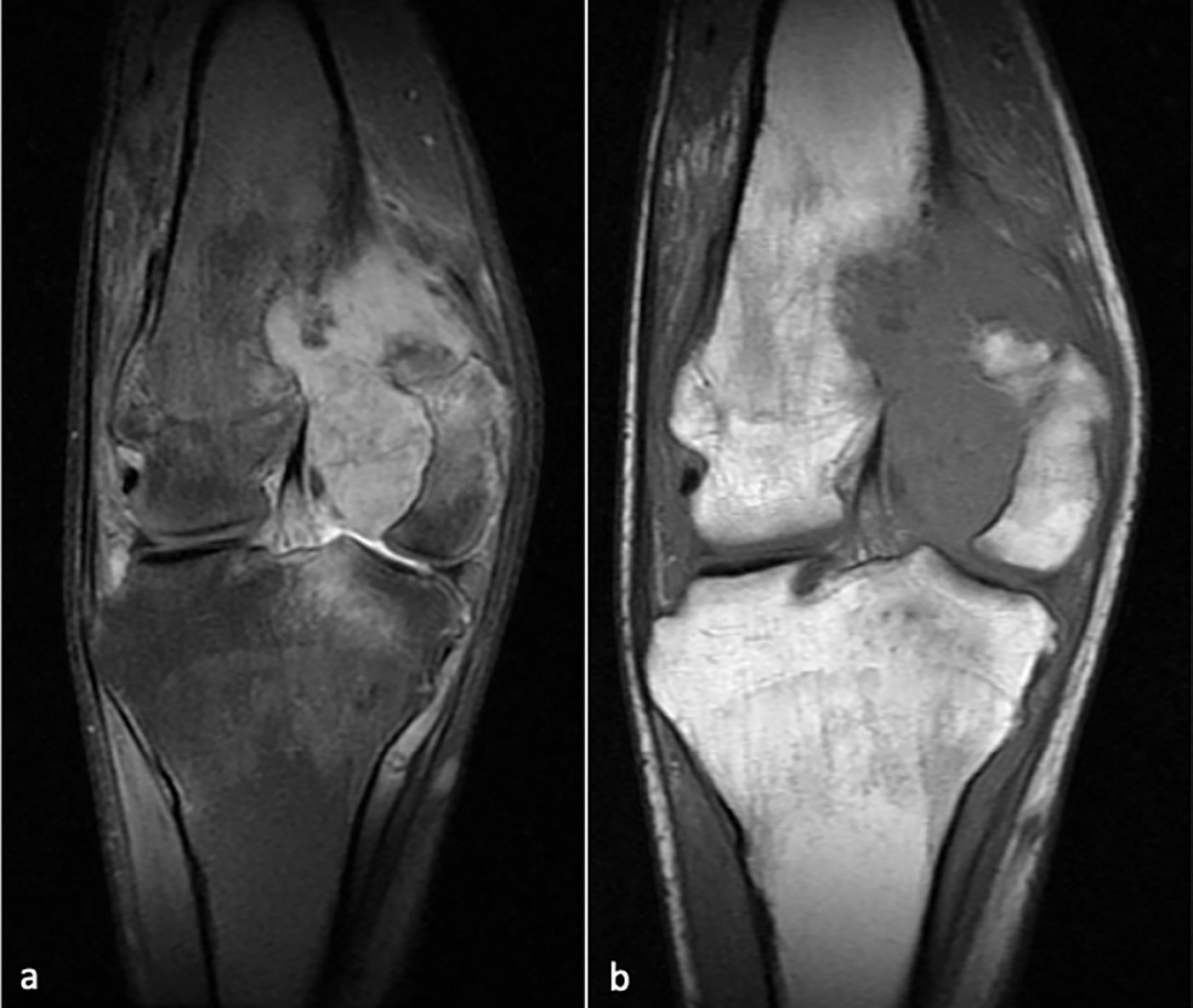
**A.** Coronal FS‑PD MRI shows a homogeneously hyperintense lobulated mass in the intercondylar groove with remodelling of the posterior medial femoral condyle. **B.** Coronal T1‑weighted MRI shows an intermediate signal mass.

**Figure 3:**
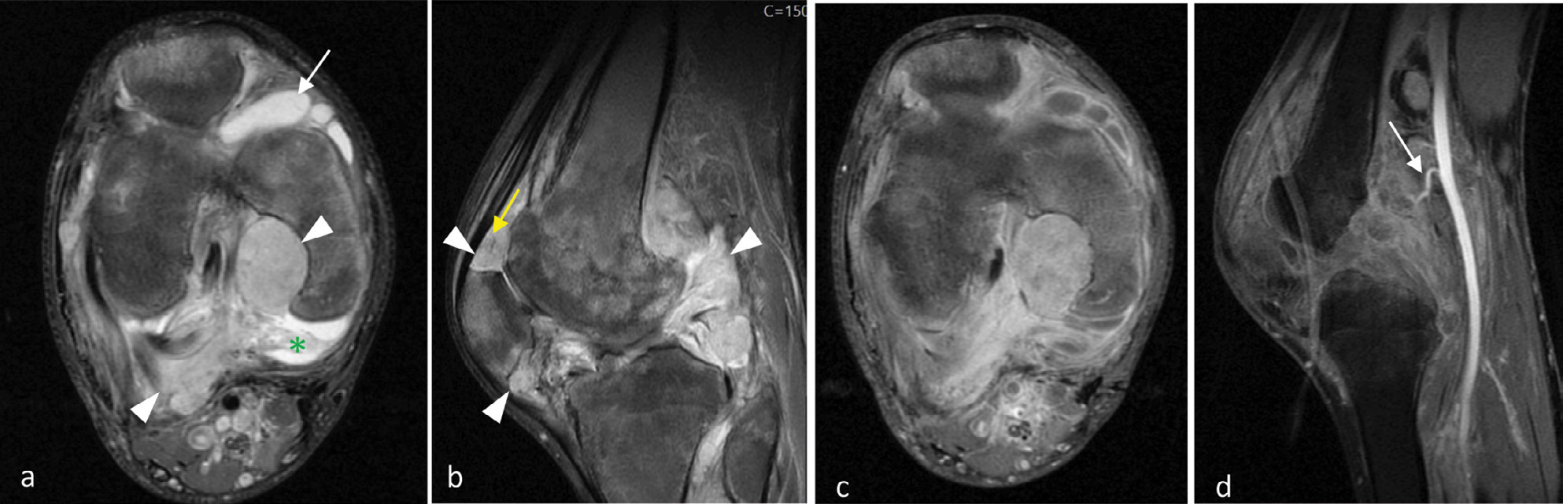
**A.** Axial FS‑PD MRI shows a lobulated mass in the intercondylar groove near the medial femoral condyle and poplitaeal fossa (arrowheads). There is joint effusion (arrow) and synovial thickening (asterisk) suggestive of reactive inflammatory changes. **B.** Sagittal FS‑PD MRI shows soft‑tissue nodules in the suprapatellar fat pad and Hoffa’s fat pad (arrowheads). Linear hypointense signals within the nodules may represent vessels with flow void (yellow arrow). **C**–**D.** Axial and sagittal T1‑weighted fat‑saturated MRI post‑gadolinium injection shows enhancement of lesions and supplying vessels from the poplitaeal artery (arrow).

**Figure 4:**
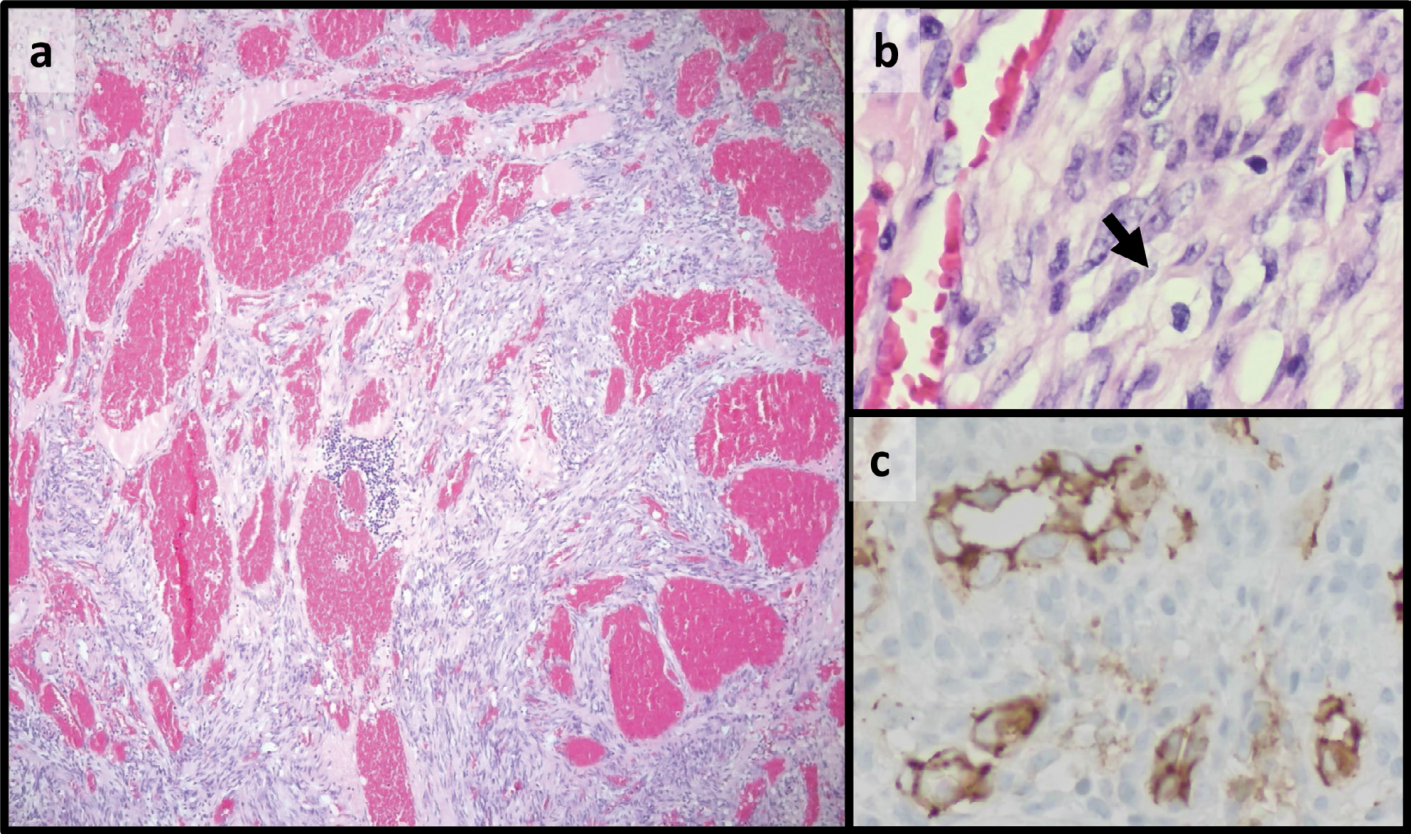
**A.** The nodules consist of proliferating dilated vessels of varying calibre among solid areas of bland, monotonous spindle‑shaped cells. **B.** Vacuolated endothelial epithelioid cells (arrow) are present, with no cytological atypia or mitotic activity. **C.** The epithelioid cells are CD31 positive. Magnification: 50x **(A)** and 400x **(B** and **C)**.

X‑ray of the knee revealed diminished bone mineralization, epiphysial overgrowth and intercondylar groove widening. In addition, degenerative changes were observed in the femorotibial compartments, including subchondral sclerosis and joint space reduction (Figure 1a‑b). Magnetic resonance imaging (MRI) revealed multiple lobulated nodular coalescent lesions within the joint space of the knee (Figure 2a–b and Figure 3a). Infiltration of the suprapatellar fat pad and Hoffa’s fat pad by the soft‑tissue masses was present (Figure 3b). These lesions were hyperintense on proton density fat‑saturated MRI sequence (FS‑PD) and showed intermediate signal intensity on T1‑weighted sequence. Significant gadolinium enhancement of the lesions (Figure 3c), with vascular supply from the poplitaeal arterial branches (Figure 3d) were obvious. Bone remodelling and secondary degenerative joint signs, such as joint space narrowing, marginal osteophytosis of the condyles and chondromalacia, were also evident. The patient underwent surgical excisional biopsy of seven nodules and histopathological examination revealed a spindle cell haemangioma (Figure 4).

## Discussion

Synovial haemangiomas can involve part or the entire synovium and are typically monoarticular. These lesions are classified on the basis of their anatomical position into synovial, juxta‑articular and intermediate types. Juxta‑articular haemangiomas are situated outside but near the joint capsule, synovial haemangiomas are located inside the joint capsule and intermediate haemangiomas are localized both inside and outside the joint capsule [1, 2]. The case presented corresponds to an intermediate type.

X‑ray features are often inconclusive, although phleboliths may be seen [1, 2]. MRI is essential for diag‑nosing synovial haemangiomas, as it determines the size and extent of the soft tissue lesions, thus largely contributing to treatment planning. SH typically appear on MRI with a lobulated shape and without significant bone destruction [3]. They lack a well‑defined capsule and may extend across tissue boundaries, remodelling anatomical structures [3]. It usually shows low to intermediate signal intensity on T1‑weighted images and high signal intensity on T2‑weighted images [2, 3]. Low‑signal intensity linear structures within the lesions on T2‑weighted images likely represent fibrofatty septa or vascular channels [2, 3].

Treatment options depend on the tumour type and extent. Localized tumours are preferably removed via arthroscopic surgery [1–3]. However, complete removal can be difficult for diffuse lesions and may necessitate an open procedure [3]. Open surgery might also be required when the lesion extends to tendons, muscles or bones, which is common in juxta‑articular or intermediate types [3]. Preoperative embolization might be required and beneficial for excision of lesions with large arterial feeding vessels [2].

## Conclusion

Synovial haemangiomas present with non‑specific clinical symptoms, characterized by recurrent joint pain and swelling, particularly in children and young adults. MRI is a crucial diagnostic technique, offering superior tissue contrast and precise delineation of the lesion. Despite its rarity, prompt recognition and accurate diagnosis are essential for optimal therapeutic assessment.
